# Impact of perceived supervisor support and leader-member exchange on employees’ intention to leave in public sector museums: A parallel mediation approach

**DOI:** 10.3389/fpsyg.2023.1131896

**Published:** 2023-03-03

**Authors:** Muhammad Asif, Mingxing Li, Abid Hussain, Arif Jameel, Weijun Hu

**Affiliations:** ^1^School of Management, Jiangsu University, Zhenjiang, China; ^2^School of Archaeology, Jilin University, Changchun, China

**Keywords:** perceived supervisor support, leader-member exchange, organizational citizenship behavior, perceived organizational support, intention to leave, museums, parallel mediation, Pakistan

## Abstract

High staff turnover in certain public sector organizations in Pakistan is a challenging problem, and organizations strive to reduce this issue using different mechanisms. Therefore, this research investigates the parallel mediation impact of perceived organizational support (POS) and organizational citizenship behavior (OCB) on the relationships among perceived supervisor support (PSS), leader-member exchange (LMX), and employee’s intention to leave (IL). Data were collected from 482 employees working in public sector museums in Pakistan in three waves. Structural equation modeling (SEM) with a two-step approach was used to evaluate the data. The research found that both POS and OCB mediate the negative relationship between PSS and IL and between LMX and IL in a parallel mediation mechanism. Public sector museums should focus on providing visible supervisory support and develop a healthy work environment where the exchange relationship between supervisors and subordinates strengthens to reduce the possibility of the employee’s leave intentions.

## 1. Introduction

Leave intentions in tourism industries, especially in the cultural/museum industry, have gained a major concern for museum administration and academicians and scholars due to its complex structure, high recruitment and training expenditure for new staff, lower productivity and less efficiency in service provision to its customers (Putra and Suana, 2016). The cultural industries have experienced high turnover for many decades. Prior research demonstrated that cultural industries significantly encountered a higher level of staff mobility and leave intention than other industries (Guzeller and Celiker, 2019; Park and Min, 2020). Since the employees in cultural tourism industries (museums) have to perform their duties for a long time, even on weekend days, their social lives are significantly affected. As a result, this will increase their job dissatisfaction, and they will turn to leave the organization. Some other important issues, such as (low wages, less social security, personal well-being etc.) have also been found in cultural industries, which reduce employees’ commitment, and they went for quit the organization. All these factors demotivated the employees, and their trust in the top organizational management was reduced (Ward et al., 2022).

Regarding GDP, Pakistan ranked 206th among 212 nations (World Population Review, 2023) and is currently dealing with several issues in the tourism sector. As a collectivistic society (Islam, 2004), it is noteworthy to observe how collectivism manifests itself when extending leadership style and supervisory support to followers and how they recognize and utilize this social support to achieve certain organizational goals. Therefore, public sector organizations in Pakistan have been facing many difficulties and challenging scenarios, including insufficient finances, poor infrastructure, lack of resources, poor policy-making and implementation, economic and political crises, and many more. The work environment in public sector organizations in Pakistan is critical. Employees are facing many hurdles, such as long working hours, low income and social security in terms of personal and family health, inadequate degree of freedom, lack of career development opportunities, low empowerment and recognition, insufficient social support, and the ability to relate their work to the larger societal equation (Zahra and Jadoon, 2016; Khan et al., 2022). Because these factors impact employee attitudes and actions, employees are more likely to depart. In line with these issues, employees working in cultural tourism industries, especially in museums, are highly influenced, and they prefer to leave the organization. Previous studies found some possible facets of employees’ leave intentions in behavioral management research, including low employee confidence and high absenteeism (Jaddoud, 2018; Burlison et al., 2021). Hence, scholars are highly focusing on the cultural tourism industry and exploring the deeper roots of the employees’ intention to leave (Jaddoud, 2018; Xu et al., 2018). Supervisory support and leader-member exchanges are critical factors in establishing strong relationships among employees in an organization (Pattnaik and Panda, 2020; Wu et al., 2021). These features enable top management to appreciate the employees’ work outcomes, performance, and well-being (Khalifa, 2019). When subordinates perceive higher support from their immediate supervisors, their interpersonal relationships are strongly developed and reciprocated; they are more satisfied with their jobs and show higher commitment to the organization (Khalifa, 2019; Mete et al., 2021). Consequently, employees’ productivity increases and lowers their intention to leave that organization, resulting in higher supervisor support and a strong exchange relationship between supervisor and subordinates (Martin et al., 2016). Perceived supervisory support (PSS) and strong leader-member exchange (LMX) have not been explored in the tourism industry, especially in the cultural tourism industry; no such relationship has been investigated to date.

Normally, museums are operated by certain government agencies/institutions and have a complex but comprehensive organizational structure with several hierarchies. Comparing other tourism industries, which mostly focus on marketing strategies, museums have a unique structure with different operational departments such as conservation, collection & research, exhibition and security, etc. (Putra and Suana, 2016). In Pakistan; museums are functionally operated by a board of governors who appoints a director and additional director to control all operations. Moreover, all heads of sub-departments are responsible for answering the additional director. The additional directional makes a team of supervisor function in different departments.

On the other hand, frontline employees work directly under the supervision of their immediate supervisor, and the supervisor is responsible for the productivity and performance of their subordinates (Rathi and Lee, 2017). In this way, the supervisors play an important role in building leader-member and organization-member relationships. As a result, the top organizational management involves the needs of its employees and provides certain benefits to them to reduce their leave intention (Alharbi and Abuelhassan, 2020). Because of a cross-level administrative ecosphere in the museum hierarchal structure, top management should seek ways to strengthen perceived organizational support (POS) at all department levels to fulfill organizational needs. Supervisors at lower organizational levels must employ certain work behaviors and perform object-oriented activities to influence supervisors who are not directly under their control (Eisenberger et al., 2014). Supervisors should then emulate the progressive efforts towards their lower-level employees by practicing actual but fair and moral responsibilities and providing necessary supervisory support to their subordinates, ensuring they express a higher organizational commitment and work engagement. As a result, the employees’ trust in their immediate supervisor and top management increases, and they feel more obliged and satisfied with their jobs, and their intention to leave the organization will be significantly decreased. When the employees feel that top management cares about their well-being, they perform well and put all efforts into achieving organizational goals (Shi et al., 2021).

Though top management frequently moves from one unit or team to another, team leaders are responsible for their coworkers’ activities and performance (Mete et al., 2021). They must manage subordinates’ tasks towards organizational goal achievement (Arici, 2018). Additionally, in museums, the provision of supervisory support and strong exchange behaviors between leaders and subordinates not only establishes a conducive environment for developing a mutually beneficial leaders-subordinate relationship but also provides opportunities for promoting strong collective trust and deepening employee job attitudes toward the museum by strengthening employee-related factors, which may reduce employee leave intention (Putra and Suana, 2016).

**Figure 1:**
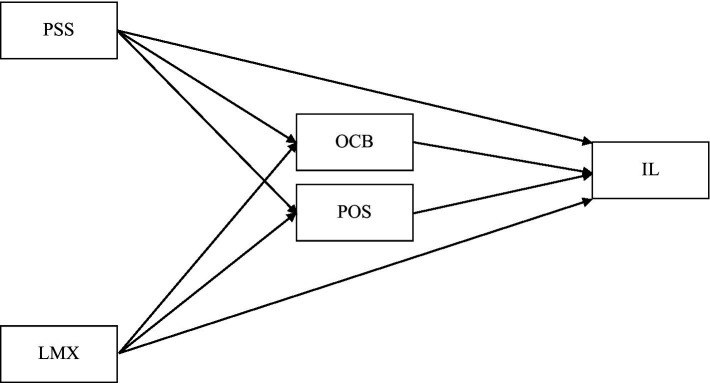
Hypothesized model.

Previously, scholars employed different factors in the relationships between PSS, LMX and IL to reduce employees’ intention to leave the organization. For example, Arici (2018) employed authentic leadership as a moderator, Chiu et al. (2020) tested the mediating and moderating effect of psychological empowerment and psychological contract breach, respectively, and Bhatnagar (2014) used reward and recognition as a mediator. To our best knowledge, no study has been found to date employing POS and OCB as potential mediators in this relationship in the tourism industry, especially museum research. We examined PSS-IL and LMX-IL relationships at the same time and used POS and OCB as parallel mediators. Our study investigates the degree to which PSS and LMX influence POS and OCB, influencing subordinates’ intention to leave the organization (Figure 1). Despite the lack of investigation, museum administration should thoroughly understand this multiple-mediation mechanism of PSS-IL and LMX-IL relationships to enhance a strong supervisor-subordinate relationship and establish a healthy work environment where the employees feel more comfortable sharing new ideas and expressing their needs.

## 2. Theoretical background and hypotheses development

### 2.1. Social exchange theory context

Social exchange theory has been considered one of the most prominent theories for understanding the relationship between employees and the organization because of its influential exchange-behavior mechanism (Cropanzano and Mitchell, 2005). Although several theorists have contributed to the theoretical framework regarding social exchange research, Blau (2017) and Alvin (1960) offered the most important influencing factors to measure these relationships. In the context of the follower-organization relationship, the social exchange theory is applied when a subordinate develops a strong association with his immediate supervisor and exchanges his ideas (Liden et al., 1997). In this way, the subordinate develops a strong relationship with the organization (Eisenberger et al., 1986; Tetteh et al., 2020). These exchange relationships enable employees to significantly contribute to achieving organizational goals, increase performance and commitment levels, and decrease turnover intention (Shore et al., 2009).

From the perspective of social exchange theory, scholars revealed that PSS relates to employees’ perception of how much value a supervisor gives them for their contribution and how often a supervisor cares about their well-being (Kottke and Sharafinski, 1988). Employees perceive their supervisor as a change agent (Eisenberger et al., 2002) and develop a strong exchange relationship that differs from those they had with their organization. Employees strongly engage in long-term interactions with their organization and supervisors (Settoon et al., 1996). As discussed earlier, employees are highly concerned about their value in the organization. They develop exchange relationships with the organizational change agents (supervisors) only when they acquire more freedom, value, and appreciation of their contribution towards goal achievement and well-being. Hence, employees maintain a balance in the exchange relationships with their organization by demonstrating positive work outcomes based on the employer’s commitment towards an individual and strong organizational support (Fukui et al., 2019). Prior research reveals that employees who perceive strong organizational support are highly committed to the organization (Burns, 2016), and their absenteeism and intentions to leave the organization significantly reduce (Eisenberger et al., 2014). Similarly, when employees experience strong supervisory support, they become more obliged to help their immediate supervisor by putting extra effort to achieve the required goals (Kurtessis et al., 2017) and voluntarily contribute their efforts to achieve additional goals which are not part of their task showing strong organizational citizenship behavior (Nadiri and Tanova, 2010). Previous studies highlight the significance of both POS and OCB as the key indications of employees’ intention to leave (Arici, 2018; Park and Min, 2020; Li and Xie, 2022) and reveal that an immediate supervisor plays a critical role in employees’ intention to leave (Lee et al., 2019; Pattnaik and Panda, 2020).

Similarly, LMX theory also provides strong evidence of subordinate-supervisor exchange relationships. Deeply associated with in-role theory (Kahn et al., 1964) and social exchange theory (Cropanzano and Mitchell, 2005; Blau, 2017), LMX theory offers a distinct concept where a supervisor forms a differential relationship with his subordinate. This differential relationship varies from low to high based on the exchange quality. For instance, subordinates who experience greater supervisory support in terms of trust have a higher level of exchange quality (Dienesch and Liden, 1986). These LMX-based high-quality exchange relationships aid the employees in different ways, such as good job-related communication, privileged treatment, provision of formal and informal incentives, direct approach to immediate supervisor, and timely performance-based feedback (Dienesch and Liden, 1986; Martin et al., 2016). Conversely, subordinates who perceived low-quality LMX relationships frequently received the opposite aid in limited supervisor’s trust and emotional support (Fanny and Sugiarto, 2022), decreasing employee performance and commitment and increasing leave intention. Based on the LMX and social exchange theories, such high-quality exchanges are associated with positive employee outcomes such as engagement, job satisfaction and affective commitment (Mete et al., 2021; Fanny and Sugiarto, 2022). More specifically, these high-quality exchange relationships are associated with those employees who enjoy special incentives, ease of supervisory access and good communication with their immediate supervisor (Gerstner and Day, 1997). All these factors enhance the employees’ behavior regarding job satisfaction and organizational commitment and reduce quit intentions.

### 2.2. PSS, OCB, and IL

Organizational citizenship behavior (OCB) is an employee commitment that involves activities that are not part of their contractual responsibilities (Organ, 1988; Nadiri and Tanova, 2010). Williams and Anderson (1991) discussed two types of OCB, individual (OCBI) and organizational (OCBO), based on socially-oriented activities. OCBI refers to an act aimed at benefiting coworkers, whereas OCBO refers to activities aimed at organizational benefits. Prior research revealed various organizational elements that affect OCBO, such as corporate moral beliefs, supportive behavior, incentive schemes, and commitment (Baker et al., 2006). Organizations that strive for a holistic and collaborative work environment are much more likely to get better OCB than those with poorer or discordant cultures (Somech and Drach-Zahavy, 2004).

Organizational support is considered one of the most significant consequences of OCB. To succeed, any organization understands the need to foster organizational support (Karatepe, 2012). Developing a cooperative team culture that emphasizes trust, empathy, fairness, and benevolence is critical to becoming a highly productive group (Ritsri and Meeprom, 2020). Employees are likely to indulge in OCB when they feel strong team support. As a result, employees are ready to invest a significant amount of effort on behalf of their members by providing novel ideas and taking initiatives that may benefit the organizational growth and progress (Tuan et al., 2021; Bekmezci et al., 2022). It further indicates that a supportive organizational structure fulfills the needs of staff consciousness, which could lead to a connection between POS and OCB, which may result in establishing employees’ voluntary behavior that leads to proper job expectations (Han et al., 2018). Another research reveals a robust link between leader cooperativeness and personnel OCB (Byun et al., 2020). As a result, when the team is cooperative and friendly, the members of that team see OCB as an important element of their job.

Several studies have discovered a significant association between OCB and employees’ productivity and a weak association between OCB and employees’ desire to resign (Ocampo et al., 2018; Ridlo et al., 2021; Li and Xie, 2022). Claudia (2018) investigates the link between organizational support, organizational citizenship behavior, and performance outcomes and indicates that increased POS leads to increased OCB, which also positively impacts job efficiency. On the other hand, Li and Xie (2022) discover a negative association between OCB and intention to leave. Furthermore, earlier research has shown that OCB offers a mediation effect in the link between work attitudes and the performance of the employees. Garg et al. (2019) investigate the function of OCB as a mediator in the link between workplace spirituality and job satisfaction. Chiang and Hsieh (2012) found OCB a potential mediator between POS and work performance in Taiwanese hotels and confirmed that a higher degree of POS among employees results in a higher OCB level, which may lead to increased employee performance organizational efficiency and decreased intention to leave the organization.

**H1:** OCB will significantly mediate the association between PSS and IL.

### 2.3. LMX, OCB, and IL

Previous studies revealed some important elements influencing OCB, including moral values, personality traits, satisfaction, and job attributes. Coherently, these elements are considered indicators of OCB (Ocampo et al., 2018; Pham et al., 2019; Purwanto et al., 2021). For instance, an employee with strong moral values shows more willingness and dedication to his work and significantly influences OCB within that organization (Götz et al., 2020; Li and Xie, 2022).

Prior research has explored the significant association between LMX and OCB (Teng et al., 2019; Sa’adah and Rijanti, 2022; Tremblay et al., 2022). LMX theory provides a strong base for leaders to develop exchange in agreements with their subordinates and appreciate and reward those who achieve the expected goals (Tremblay et al., 2022). Several empirical studies have shown that LMX significantly impacts employee work outcome attitudes, including job satisfaction, organizational justice, autonomy, affective commitment, role clarity, competency, and performance (Eisenberger et al., 2014; Martin et al., 2016; Teng et al., 2019). Furthermore, a high-quality LMX connection predicts employee OCB, fostering mutual support and a sense of altruism and job commitment among employees (Fanny and Sugiarto, 2022). Moreover, Teng et al. (2019) confirmed the favorable relationship between LMX, OCB and employee performance. Previous studies also confirmed a significantly positive association between LMX and employee performance, while a significantly negative association between LMX and intention to leave (Teng et al., 2019; Li and Xie, 2022). Sun et al. (2007) investigated service-oriented OCB as the potential mediation between high-performance HR practices and productivity. They also highly recommended performing more research into the mediating role of OCB in the relationship between employee attitude and other organizational performance indicators. As a result, we hypothesize that OCB acts as a mediating variable between LMX and the employee’s intention to leave.

**H2:** OCB will significantly mediate the association between LMX and IL.

### 2.4. PSS, POS, and IL

Scholars referred to PSS by arguing that employees have broad opinions about how often their superiors are concerned with individual well-being and appreciate and reward them based on their contributions towards goal achievement (Rathi and Lee, 2017). Existing literature shows that PSS received higher attention in the organizational context and is significantly associated with job-related attitudes (i.e., intention to leave; Lee et al., 2019; Pattnaik and Panda, 2020). Research also indicates a significant positive association between PSS and POS (Burns, 2016; Kurtessis et al., 2017). Levinson (2009) argued that the Friendly behavior of a supervisor might enhance employees’ level of POS because of the organizational attributes directly like it. Since the supervisor acts as an organizational agent and fulfills the responsibilities to direct their subordinates and evaluate their performance; as a result, the employee believes this positive orientation from their superior as an organizational support (Pattnaik and Panda, 2020; Piotrowski et al., 2021). When a supervisor properly evaluates the performance of subordinates, conveys the top management, and re-communicates top management’s views to the subordinates, it enhances the relationship between supervisor and subordinates, which results in strengthening the linkage between PSS and POS (Burns, 2016). Several studies found a relationship between PSS and POS in different contexts. For example, Alkhateri et al. (2018) found that PSS impacts employee attitudes (i.e., job satisfaction, commitment and turnover intention) with a full mediation approach. While in another study, Fukui et al. (2019) discovered a full mediation of burnout and job satisfaction between supervisory support and leave intention. Similar claims are also confirmed by other scholars (Mathieu et al., 2016; Rathi and Lee, 2017). Becker (1992) argued that employees might differentiate their relationships with their supervisors and with their current organization. Supervisor characteristics could explain employee behavior differences regarding the intention to leave and commitment. These findings clearly show that PSS should result in attachments to the supervisor and the organization. Based on these results, we hypothesized;

**H3:** POS will significantly mediate the association between PSS and IL.

### 2.5. LMX, POS, and IL

Employees are highly concerned about their contribution and the degree of POS, owing to the organization’s clear willingness to recognize more employees’ efforts in attaining organizational goals. Social exchange theory suggests that leaders and organizations motivate workers to utilize their resources, talents and time (Blau, 2017). Employees believe that a possible gain in terms of both material and symbolic benefits may result from higher job performance and commitment to achieving the organizational goals (Eisenberger et al., 2014; Andersen et al., 2020). As a result, both POS and LMX are the key elements influencing individuals’ motivation. The previous study found both positive and negative impacts of LMX on employees’ job-related attitudes (Wang et al., 2018). LMX significantly enhances positive reciprocal behaviors, such as OCB (Martin et al., 2016), and strongly influences employees’ IL (Dulebohn et al., 2012). Similarly, in the Chinese context, the term “Guanxi” strongly ties the supervisor-subordinate relationship on the bases of social exchanges (Mejia et al., 2020). Such relationships generally involve job-related activities and strongly influence employee attitudes.

On the other hand, scholars found that POS strongly influences employees’ work outcomes, including commitment and intention to leave (Van Knippenberg and Sleebos, 2006). Moorman et al. (1998) explored a strong association between POS and OCB. He also found that POS can influence multi-dimensional behaviors of employees, such as interpersonal assistance, professionalism, and enthusiastic boosterism. Furthermore, empirical research suggests a slight association between POS and employees’ withdrawal behavior (Eder and Eisenberger, 2008). Intention to leave (IL) is a degree of an individual’s willingness to leave the organization (Mobley, 1977). Since IL is an important work attitude in the organizational context, it directly affects the organization and society and is an important factor in future research. In previous studies, scholars found several components that significantly influence IL. These components include an organization’s structure, financial and social aspects, external and internal environment and psychological manners (Gable et al., 2007; Eby et al., 2010). Based on the existing research, scholars developed various assumptions to test the possible causes of IL and divided them into three groups (i.e., job-related content, individual perception, and organization environment; Daly and Dee, 2006; Eby et al., 2010; Haines et al., 2010). As a result, it enables the leaders to decide the rewards for recognition of subordinates and helps them to prevent the loss-ratio of productivity. Similar to the above literature, we analyze the association between employees’ perceptions of IL. In this research, our main concern is to explore the association between leaders-member, perceived organizational support and employees’ intention to leave *via* a mediation mechanism.

**H4:** POS will significantly mediate the association between LMX and IL.

## 3. Method

### 3.1. Procedure

Pakistan has a wide range of public sector museums that offer visitors various information. For instance, archaeology museums display archeological artifacts; art museums show visual art objects such as paintings, sculpture, photography, etc.; history museums possess a collection of artifacts that tell a chronological story about a particular locality, natural history, dinosaurs, zoology, oceanography, anthropology, evolution and museums devoted to science and technology (Department of Archaeology and Museums, 2023). In this research, we randomly chose six public sector history museums from different cities in Punjab province. Among them, three museums were in Lahore, and the remaining three were in Bahawalpur, Multan and Faisalabad. All six museums have a similar rich historical background to each other. The study adopted a convenience sampling method to collect data (Liu et al., 2015). Self-administered questionnaires were used to collect data from the employees (subordinates and immediate supervisors) working in public sector museums in Punjab Province (Pakistan). We directly approached the HR department, explained the study’s nature and procedure, and clarified their voluntary participation. We assured all respondents about the confidentiality of the data and the responses provided by them. Following the recommendations of Podsakoff et al. (2012), we obtained data from the respondents (subordinates and supervisors) at three different times with a 4-week interval to reduce the possibility of common method bias.

Additionally, we used the Marker Variable technique recommended by Lindell and Whitney (2001)) by inserting a theoretically un-related variable, “Machiavellianism” developed by Christie and Geis (1970), with a sample item “The best way to handle people is to tell them what they want to hear” in correlations table and found no possibility of common method variance (CMV; Table 1). We collected data from multi-sources (i.e., supervisors-subordinates) at three different times, which significantly enhanced the validity of the data by corroborating time-based separation and overcoming the key limitation of investigating causal relationships. We obtained data for demographic details and independent variables (i.e., PSS and LMX) from the respondents at time one, and the data for mediating variables (OCB and POS) were obtained at time 2. Finally, the dependent variable (IL) data were collected at Time 3. We used codes to match the responses of the subordinates and their immediate supervisors. We distributed the questionnaire to 651 respondents and received 482 completed responses (based on supervisor-subordinate). The sample characteristics reveal that most respondents are in the age range of 20–29 (43.36%). The number of female respondents (11.0%) is much lower than that of male respondents (89.0%). In terms of the level of education, the majority of respondents are graduates (69.92%). In the study, lower-level employees (70.95%) dominate the sample, as presented in Table 2.

**Table 1:** Descriptive statistics.

*N* (482)	Mean	SD	Correlations									
			1	2	3	4	5	6	7	8	9	10
AVE	-	-	-	-	-	-	0.77	0.80	0.79	0.67	0.71	0.69
1. Age	33.12	10.85	-									
2. Gender	1.29	0.46	0.01	-								
3. Education	1.89	0.43	−0.08	0.02	-							
4. Position	4.75	3.67	0.13*	0.16**	−0.12*	-						
5. PSS	4.22	0.59	0.11*	0.09*	−0.06	0.08	-					
6. LMX	3.91	0.73	−0.05	0.11*	0.09	0.13*	0.21**	-				
7. OCB	2.15	0.67	−0.08	0.01	0.01	−0.05	0.38**	0.28**	-			
8. POS	3.17	0.97	0.18**	0.14**	0.06	0.06	0.29**	0.34**	0.25**	-		
9. IL	2.68	0.72	−0.10	0.03	0.01	0.04	−0.41**	−0.24**	−0.38**	−0.29**	-	
10. Machiavellianism	2.18	0.58	−0.07	0.01	0.05	0.03	−0.11*	−0.03	0.00	−0.06	0.04	-

*p < 0.05; **p < 0.01.

**Table 2:** Demographics details.

Description	No.	Percentage
Gender		
Male	429	89.00
Female	53	11.00
Age		
20–29	209	43.36
30–39	153	31.74
40–49	92	19.09
≥50	28	5.81
Education		
Intermediate	25	5.19
Graduate	337	69.92
Master	120	24.89
Position		
Non-managerial	342	70.95
Managerial	140	29.05

### 3.2. Measures

We used a 5-item Likert scale (1 for “strongly disagree” and 5 for “strongly agree.”) to measure the variables. Other variables’ details are provided below:

#### 3.2.1. Intention To leave

The data for IL is obtained using a 3-items scale established by Colarelli (1984). The sample question for this measure is “I frequently think of quitting my job,” with an alpha reliability of 0.86.

#### 3.2.2. Perceived supervisor support

House (1981) developed the 5-items PSS measure (1981). The sample question for this measure is “My supervisor is willing to listen to my work-related problems,” with an alpha reliability of 0.78.

#### 3.2.3. Perceived organization support

An 8-items POS scale was developed by Eisenberger et al. (1986). The sample question for this measure is, “My organization really cares about my well-being,” with an alpha reliability of 0.89.

#### 3.2.4. Organizational citizenship behaviors

We adopted a 3-item scale to measure OCB developed by Liden et al. (2004). The sample question for this measure is, “This subordinate volunteer to do things not formally required by the job,” with an alpha reliability of 0.77.

#### 3.2.5. Leader-member exchange

LMX scale included 8 items and was developed by Bauer and Green (1996). The sample question for this measure is “How would you characterize your working relationship with your leader?” with an alpha reliability of 0.83.

## 4. Data analysis

We employed a structural equation modeling technique to test our study hypotheses *via* AMOS 25.0. We followed Anderson and Gerbing (1988) recommendation of a 2-step SEM approach where we first performed confirmatory factor analysis (CFA) for model appropriateness. After that, a final hypothesized structural model was analyzed to test the relationships among all variables. Several fit indices (Kanwel et al., 2019; Manzoor et al., 2021), including χ^2^/df, comparative fit index (CFI), standardized root mean square residual (SRMR), Tucker-Lewis index (TLI), and root mean square error of approximation (RMSEA), were employed while performing CFA. Descriptive statistics, including correlations among all variables, means and standard deviations, are provided in Table 1.

### 4.1. CFA and measurement model assessment

We measure four different models using five studied variables in CFA to confirm the acceptability of our theoretical model. The results in Table 3 revealed that the 5-factor model is the best fitted compared to other alternative models where we measured all variables, including PSS, LMX, POS, OCB and IL. Values for 5-factor fitness model were χ^2^ = 338.21, df = 219, χ^2^/df = 1.54, CFI = 0.96, TLI = 0.95, RMSEA = 0.04, SRMR = 0.04. As West, Taylor (Bauer and Green, 1996) recommended, these values provide appropriate cutoff criteria. On the other hand, we measured two alternative 4-factor models by combining PSS and LMX (χ^2^ = 689.35, df = 223, χ^2^/df = 3.09, CFI = 0.88, TLI = 0.89, RMSEA = 0.08, SRMR = 0.09) and POS and OCB (χ^2^ = 857.12, df = 225, χ^2^/df = 3.81, CFI = 0.78, TLI = 0.78, RMSEA = 0.10, SRMR = 0.12). Alternatively, a single-factor model was also measured (χ^2^ = 1044.85, df = 228, χ^2^/df = 4.58, CFI = 0.65, TLI = 0.64, RMSEA = 0.15, SRMR = 0.14) in CFA. These results provide the discriminant validity of our model and reduce the possibility of common method variance (CMV; Podsakoff et al., 2003, 2012).

**Table 3:** CFA Results.

Model	χ^2^	Df	χ^2^ /df	Δχ^2^ (Δ df)	CFI	TLI	RMSEA	SRMR
5-factor model	338.21	219	1.54	-	0.96	0.95	0.04	0.04
4-factor model (PSS & LMX combined)	689.35	223	3.09	351.14 (4)	0.88	0.89	0.08	0.09
4-factor model (POS & OCB combined)	857.12	225	3.81	518.19 (6)	0.78	0.78	0.10	0.12
1-factor model	1044.85	228	4.58	706.64 (9)	0.65	0.64	0.15	0.14

We also measured internal consistency using Cronbach’s Alpha, convergent validity, and composite reliability CR. As shown in Table 4, the alpha reliabilities ranged from 0.77 to 0.89 for each variable which is statistically significant as recommended in previous literature (Nunnally, 1994; Manzoor et al., 2021). Similarly, we found significant CR results ranging from 0.82 to 0.91, as recommended by Bagozzi and Yi (2012) and Fornell and Larcker (1981). Factor loadings are provided in Table 4, which range from 0.71 to 0.87 and are greater than the recommended criterion value of 0.50 (Hwang et al., 2020; Hair et al., 2022), showing convergent validity for our 5-factor model. Values of average variance extracted (AVE) are given in Table 1. These values are greater than 0.5 and provide convergent validity (Fornell and Larcker, 1981).

**Table 4:** Measurement model.

Factor	Items	Loadings	S.E.	T	C.R.	α
Perceived supervisor support	PSS1	0.76	-	-	0.82	0.78
	PSS2	0.83	0.052	15.96**		
	PSS3	0.82	0.056	14.64**		
	PSS4	0.78	0.051	15.29**		
	PSS5	0.80	0.046	17.39**		
Leader-member exchange	LMX1	0.79	-	-	0.86	0.83
	LMX2	0.76	0.048	15.83**		
	LMX3	0.81	0.046	17.61**		
	LMX4	0.86	0.060	14.33**		
	LMX5	0.81	0.057	14.21**		
	LMX6	0.79	0.049	16.12**		
	LMX7	0.76	0.049	15.51**		
	LMX8	0.81	0.057	14.21**		
Organization citizenship behavior	OCB1	0.83	-	-	0.82	0.77
	OCB2	0.77	0.042	18.33**		
	OCB3	0.79	0.058	13.62**		
Perceived organizational support	POS1	0.82	-	-	0.91	0.89
	POS2	0.78	0.052	15.00**		
	POS3	0.87	0.048	18.12**		
	POS4	0.82	0.045	18.22**		
	POS5	0.86	0.059	14.57**		
	POS6	0.71	0.046	15.43**		
	POS7	0.75	0.049	15.30**		
	POS8	0.73	0.044	16.59**		
Intention to leave	IL1	0.86	-	-	0.85	0.86
	IL2	0.78	0.042	18.57**		
	IL3	0.80	0.044	18.18**		

**p < 0.01.

### 4.2. Hypothesis testing

**Figure 2:**
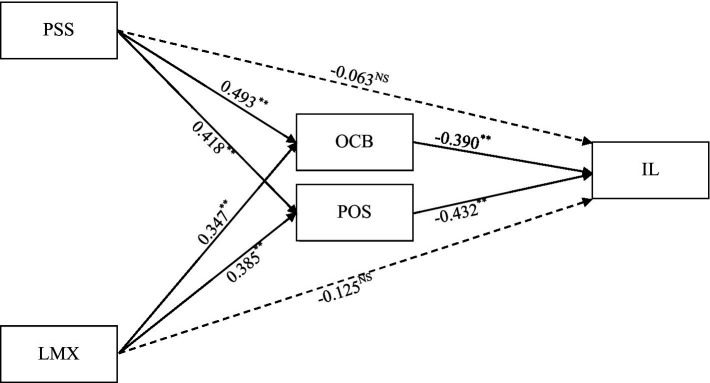
Structural model.

Our hypothesized structural model for final hypotheses testing is based on CFA’s results, which deal with a 5-factor model with the best-fit indices. As discussed earlier, the 5-factor model demonstrated the best-fit measure; we calculated SEM results using AMOS 25.0 with 95% bootstrapping confidence interval (CI). T-values for each hypothesis show significance with cutoff criteria of 1.96 (Kim et al., 2019). Hypothesis 1 of our study, reveals that ‘OCB will significantly mediate the association between PSS and IL.’ It can be seen in Table 5 that the β coefficient from PSS and IL becomes insignificant (β = −0.063; S.E. = 0.061; *t* = −1.033; CI = −0.163, 0.224) in the presence of OCB, while the indirect beta coefficient has a significant value (β = −0.193; S.E. = 0.058; *t* = −3.328; CI = −0.279, −0.493). These results show that OCB mediates the relationship between PS and IL. Similarly, hypothesis 2 reveals that OCB mediates the relationship between LMX and IL. Table 5 shows that the beta coefficient’s value is significant, showing a significant mediation mechanism. The indirect relationship for hypothesis 2 is significant (β = −0.138; S.E. = 0.063; *t* = −2.190; CI = −0.283, −0.439). On the other hand, the indirect effect of POS on the relationships between PSS and IL and between LMX and IL is significant, presenting significant mediation and supporting hypotheses 3 and 4. For instance, the indirect effect for hypothesis 3 provides significant values such as (β = −0.187; S.E. = 0.062; *t* = −3.016; CI = −0.195, −0.348; see Table 5; Figure 2). Similarly, Table 5 shows a significant indirect effect for hypothesis 4 (β = −0.163; S.E. = 0.059; *t* = −2.763; CI = −0.189, −0.437).

**Table 5:** Direct and indirect β coefficients.

	β	S.E.	*t*	Bootstrapping 95% confidence interval	
				Lower limit	Upper limit
Direct effects					
PSS IL (In presence of mediator)	−0.063	0.061	−1.033	−0.163	0.224
LMX IL (In presence of mediator)	−0.125	0.085	−1.471	−0.110	0.005
PSS OCB	0.493	0.058	8.500	0.197	0.472
LMX OCB	0.347	0.059	5.881	0.453	0.686
PSS POS	0.418	0.065	6.431	0.269	0.610
LMX POS	0.385	0.073	5.274	0.348	0.529
OCB IL	−0.390	0.062	−6.290	0.215	0.537
POS IL	−0.432	0.059	−7.322	0.198	0.473
Indirect effects					
PSS OCB IL	−0.193	0.058	−3.328	−0.279	−0.493
LMX OCB IL	−0.138	0.063	−2.190	−0.283	−0.439
PSS POS IL	−0.187	0.062	−3.016	−0.195	−0.348
LMX POS IL	−0.163	0.059	−2.763	−0.189	−0.437

## 5. Discussion

This research investigates the mediating mechanism of POS and OCB on the links between PSS and IL and LMX and IL in the museum sector. Our findings suggest that POS and OCB both significantly mediate these associations. First, the link between PSS, POS, and IL was initially investigated in this study. Moreover, the negative association between PSS and IL was shown to be significantly mediated by POS (β = −0.187; S.E. = 0.062; *t* = −3.016; CI = −0.195, −0.348). These results are consistent with the concept that PSS establishes obligations on employees to respond reciprocally by committing to the organization, resulting in decreased IL. These results align with prior research conducted in Western countries (Fornell and Larcker, 1981; Arici, 2018) and empirically investigated in an Asian context (Cheng et al., 2015; Afzal et al., 2019), revealing a vital association between POS and organizational outcomes. Secondly, our study results demonstrated that OCB significantly mediated the direct association between PSS and IL (β = −0.193; S.E. = 0.058; *t* = −3.328; CI = −0.279, −0.493). These results are similar to prior findings (Tremblay and Gibson, 2016; Tremblay et al., 2022), supporting the hypothesis that employees develop strong affiliations and exhibit positive attitudes toward their immediate supervisors, which are quite different from their specific attitudes toward the organization. In other words, supervisors perform a duty of a change agent between employees and the organization by providing adequate support to their immediate subordinates. Our research findings indicate the importance of supervisor support in tourism because of its unique organizational culture, which develops a close bond between leaders and subordinates.

Our results further demonstrated that POS and OCB significantly mediate the direct association between LMX and IL. The results suggest that both POS and OCB replaced the impact of LMX on IL *via* mediating mechanism. For instance, in any organizational context, supervisors originate and communicate goal-oriented information, rules and regulations, and the procedures required to achieve those organizational goals to their subordinates directly, increasing the supervisor-subordinate exposure and resulting in strong interpersonal relationships. In this way, the employees feel more obligation towards the organization, and supervisors play a role of a bridge between the employees and the organization (Eisenberger et al., 2014). Hence, LMX has been regarded as a significant element influencing employees’ POS and OCB, which decreases the employee’s propensity to quit the firm. In our study, we found statistically significant indirect effects from LMX to IL *via* POS (β = −0.163; S.E. = 0.059; *t* = −2.763; CI = −0.189, −0.437) and OCB (β = −0.138; S.E. = 0.063; *t* = −2.190; CI = −0.283, −0.439), while the insignificant direct relationship between LMX and IL (Figure 2). In other words, POS and OCB play an important mediating role in the association between LMX and IL. Although there is an indirect link from LMX to IL, our results illustrate the importance of supervisors’ empathy and regular support perceived by subordinates, which may be encouraged and supported in the organization. Our research further reveals how leaders influence their subordinates and set an example for top management to strengthen cooperation between superiors and followers through overall organizational beliefs and norms (Tavares et al., 2016). Our findings signify that an immediate supervisor is a person who potentially influences subordinates’ behavior and symbolizes the organization.

### 5.1. Theoretical implications

This research employed public sector museum employees and developed a multiple-median approach to determine how perceived organization support (PSS) and leader-member exchange (LMX) trickles down the public sector museum employees to influence perceived organization support (POS) and organizational citizenship behavior (OCB), which ultimately influences frontline employees’ intentions to leave (IL) the organization.

Prior research suggested that scholars conduct more research evaluating the multiple mediation causal effects within the organization to examine the employees’ work behaviors (Zhu et al., 2019; Gan et al., 2020). Beyond the traditional ways to evaluate the employees’ work-related attitudes, this research employed a multiple-mediation approach to explain the causal relationships of POS and OCB of public sector museums. Although the research organization structure is complex, this research approach is coherent with previous findings. For instance, Fukui et al. (2019) claim that supervisors often formulate their subordinates’ behaviors, which are not targeted toward a specific group of employees but toward mutual goal achievement within the organization.

Our findings offer novel insight into OCB and POS and their intervening effects on the association between PSS, LMX and IL on museum employees. Previous studies found a significant direct link between PSS, LMX and POS and OCB and a negative association between OCB, POS and employees’ IL. On the other hand, how PSS and LMX indirectly affect employee IL has not been examined thoroughly. OCB and POS received very little attention as intervening variables in reducing the employees’ IL. Our study findings prove that a significant increase in PSS and LMX can incite the level of POS and OCB among museum employees, resulting in increased performance and reduced IL.

This study proposed a multiple-mediation relationship using OCB and POS as potential mediators. Previous studies corroborate that PSS is significantly associated with individual employees’ attitudes and IL. However, our findings provide statistically significant mediating roles of both POS and OCB. For instance, we noticed that POS mediates the PSS-employee IL relationship, which ultimately affects museum employees’ IL and reduces it. On the other hand, PSS positively influences employees’ POS, which helps the top management reduce their employees’ IL.

Additionally, we found a strong effect of PSS on employees’ IL *via* mediating effect of POS. These results reveal that museum employees with a greater sense of POS show less willingness to leave the organization. Similarly, PSS indirectly affects employees’ sense of leaving the organization through OCB, which shows employees feel more comfortable with their supervisors’ behavior and ultimately feel more committed to the organization (Tetteh et al., 2020). Hence, the predictive effect of PSS on museum employees’ IL can offer enhanced precision after exhibiting the mediating effects of OCB.

Our findings confirmed a complete mediation effect of POS between PSS and IL and LMX and IL relation, proposing that employees who enjoy continuous support from their immediate supervisors and have strong interpersonal relationships sense greater support from their organization. Conversely, no expected research evidence was found of a direct association between PSS, LMX, and IL. Our findings align with Lee et al. (2019), who argued that public sector employees perceive their colleagues as a distinct commitment pivot compared with their organization. In other words, individuals view strong relationships and support (i.e., pro-social support) from their supervisors as directly descending from the organization itself.

We explored the complete mediating effects of OCB on both PSS-IL and LMX-IL relationships. These findings are consistent with previous research, which argues that OCB makes the employees more satisfied and, reciprocally, they show more commitment to their organization which, in other words, reduces the IL. Our research also supports the idea that employees develop unique ties and positive attitudes toward their leaders, which distinctly differ from their attitudes toward their organization. Supervisors aid their subordinates in two-way communication by working as organizational agents.

### 5.2. Practical implications

Our study has significant implications for the museum industry in boosting employee OCB and POS and lowering IL. The results show that boosting the level of LMX and PSS may enhance employees’ OCB and POS perception, ultimately reducing leaving intention. To encourage employee retention and loyalty, government needs to improve and offer clear but visible support to their workers including recreational facilities, social security in terms of health, working-hours flexibility, health insurance of family, etc. It is necessary for the government to design these facilities based on the needs and requirements of the workers in order to promote and enhance his retention behavior (Malek et al., 2018; Pattnaik and Panda, 2020). In addition, government should also focus on the resource allocation for generating above-mentioned facilities and should take care of wasting the resources while maximizing and improving employee OCB and POS.

Strengthening PSS and LMX for any organization is less expensive and practically measurable than other employees’ performance indicators, such as remuneration, employee training, and professional development. It is important for the Pakistani government to create a healthy environment if public sector museum where the supervisors feel more courageous to present themselves as an agent of the particular organization in order to become a main source of organizational support, resulting in increased POS and OCB and directly reducing employee IL. For example, leaders may be urged to meet regularly with their subordinates and explore what practical support they need from the organization to improve their job satisfaction and performance. However, in some situations, enhancing supervisor support may harm the organization. For example, when a subordinate receives greater support from their immediate supervisor, his affiliation or behavioral attachment will be stronger toward the supervisor than that organization. In other words, employee actual IL will increase when the immediate supervisor quits (Arici, 2018). To tackle this situation, public sector organization in Pakistan such as museums should evaluate supervisors’ performance properly and reward them accordingly for retaining their supervisory support, which helps the organization be more productive.

Government should make the policies which offer greater emphasis to the employee support in fostering POS and OCB and reducing leave intention. Our study findings suggest a negative association between an individual’s PSS and LMX and their IL, which is mediated through OCB and POS. it is important for the government to concentrate the important roles of the immediate supervisors to enhance the levels of POS and OCB for decreasing quit intention. The study’s findings suggest a negative association between an individual’s PSS and LMX and their IL, which is mediated through OCB and POS. To promote staff retention, public sector museums in Pakistan should find the ways to increase supervisory support and exchange relationships between supervisors and subordinates in the workplace in terms of non-formal mentoring, which acknowledges subordinates to build strong relationships (Malek et al., 2018).

The Government of Pakistan should endeavor to establish a culture enabling employees from different branches to interact at different levels. This goal can be achieved only by organizing sessions where the employees freely communicate with each other and share their ideas and views about their work and organizational goals. As a result, it may encourage employees to develop more productive teams, and their intention to leave the organization will be reduced.

For long-term relationships between employees, coworker support could be a better way to retain the employees. When a coworker quits the organization, it will not have the same influence on an individual’s POS as when a supervisor quits the organization. Hence, in the future, empirical research may examine this assumption by undertaking longitudinal research to test the long-run influence of coworker support on the PSS-IL and LMX-IL relationship by utilizing various multiple-mediation approaches.

### 5.3. Study limitations and future research

Two main limitations exist in our study. Firstly, the results of our study are generalizable to the same research population (e.g., employees working in public sector museums in Pakistan). Additional replication could be conducted in other tourism sectors to get more fruitful and generalized results. Secondly, our study used cross-sectional data, which objectively lacks to measure the actual leave intention of the employees at a certain time point which means it is complicated to evaluate the data causality. However, existing literature reveals that the intention to leave is deeply linked to the actual leave behavior, significantly lessening this issue.

Considering the above limitations, our study supported the findings of prior research conducted in Western countries by utilizing different factors influencing employees’ intention to leave (Madden et al., 2015; Mathieu et al., 2016). Moreover, this is the first research in which we examined the impact of PSS and LMX on employees’ intention to leave simultaneously, where we employed two potential parallel mediators (OCB and POS) simultaneously and found full mediation effects.

## Data availability statement

The raw data supporting the conclusions of this article will be made available by the authors, without undue reservation.

## Ethics statement

The studies involving human participants were reviewed and approved by the ethics committee at Jiangsu University, China. The patients/participants provided their written informed consent to participate in this study.

## Author contributions

MA wrote the first draft of the theoretical background and conducted the data analysis and wrote the first version of the findings section of the manuscript. MA, AH, and AJ contributed to the study’s design and collected the data. ML and WH supervised the study and contributed to further developing the findings section. All authors contributed to the article and approved the submitted version.

## Funding

This research is supported by the Jiangsu Outstanding Postdoctoral Program (2022ZB646), Philosophy and Social Science Research Innovation Team Project at Jilin University (No. 2022CXTD17), and the Social Science Foundation of Jilin Province (2022B101220).

## Conflict of interest

The authors declare that the research was conducted in the absence of any commercial or financial relationships that could be construed as a potential conflict of interest.

## Publisher’s note

All claims expressed in this article are solely those of the authors and do not necessarily represent those of their affiliated organizations, or those of the publisher, the editors and the reviewers. Any product that may be evaluated in this article, or claim that may be made by its manufacturer, is not guaranteed or endorsed by the publisher.
